# Oral Cancer Knowledge Assessment: Newly Graduated versus Senior Dental Clinicians

**DOI:** 10.1155/2018/9368918

**Published:** 2018-02-14

**Authors:** Gisele Pavão Spaulonci, Ricardo Salgado de Souza, Vanessa Gallego Arias Pecorari, Luciano Lauria Dib

**Affiliations:** Paulista University (UNIP), Indianópolis, SP, Brazil

## Abstract

The present study assessed the level of dentists' knowledge regarding oral cancer in the city of São Paulo, Brazil. A questionnaire was used to compare the level of knowledge among newly graduated and senior clinicians. A total of 20,154 e-mails were correctly delivered to the dentists registered in the database of the Regional Dentistry Council of São Paulo, and 477 (2.36%) responses were received. This sample consisted of 84 newly graduated clinicians and 105 senior clinicians. For the statistical analysis, the chi-square test and the logistic regression analysis were performed with *α* = 0.05, and the results were described herein. According to their knowledge level, the results were statistically different between the groups, since 19% of the newly graduated clinicians were evaluated with knowledge grade A (excellent) in comparison to 6.7% of the senior clinicians. In spite of the results indicated that newly graduated clinicians' knowledge regarding oral cancer was 2.1 times higher, 34.5% of the professionals in this group had regular or poor knowledge on the subject, and several questions relating to clinical characteristics and risk factors indicated that there still exist some knowledge gaps, demonstrating that there is a need for further studies and information activities addressing oral cancer.

## 1. Introduction

Oral cancer is considered a worldwide public health problem. It is the sixth most frequent type of cancer, and two out of three cases occur in developing countries [1, 2]. The Brazilian National Cancer Institute (INCA) had estimated the occurrence of 11,140 new cases of oral cancer in men and 4,350 in women in 2016 [3]. According to this estimation, the south and southeast regions would be the most affected, with the highest incidence rates [3].

The most prevalent type of cancer is the squamous cell carcinoma [3–5]. It is considered to have poor prognosis [6], with a five-year survival rate in 50 to 60% of cases [1, 6, 7]. It is worth mentioning that there has not been notable prognosis improvement in the recent decades [5–7].

The patients' survival rate and the functional consequences are related to the disease staging at the time of diagnosis [8]. The early detection and the immediate treatment of oral cancer may reduce the mortality rates [1, 2, 5, 9–11]. However, studies have demonstrated that two out of three cancers are diagnosed in advanced stages (III and IV) [2, 10, 12–14]. This delay in diagnosis is due to factors related to patients [13–15], health profissionais [13, 14], and the health system, as the late diagnosis has also been associated with the difficult access to specialized services, especially for people who live away from large centers [15].

The shortage of dental professionals and dental schools in Brazil may be ruled out as a possible factor for this delay [16], given that there are currently more than 280,000 professionals and 220 dental schools in the country [17].

The oral cancer may be identified at an early stage by means of visual and tactile examinations, and the dentists are key role health professionals in counselling patients about early detection of this disease [18]. The preventive role of these professionals relies on the fact that they have the greatest chances to identify asymptomatic lesions through routine examinations and to diagnose the disease before it starts unfolding, revealing its devastating consequences [19]. This fact emphasizes the importance of assessing the clinical professional knowledge regarding oral cancer risk factors and its diagnostic procedures.

Studies have been carried out in several parts of the world, indicating dentists' poor or lacking knowledge regarding oral cancer [2, 14, 18, 20–25]. In Brazil, some studies have been performed using a questionnaire previously published in the study of Dib et al. [19], which, at that time, demonstrated the low level of professional knowledge on this theme [8, 26–29].

Previous studies in countries like Yemen and Canada have suggested that newly graduated professionals had more knowledge in comparison to senior professionals [4, 25]. The hypothesis of the present study is that there may be differences in dentists' knowledge due to the number of years of professional experience or seniority. However, there are doubts whether newly graduated dentists have more knowledge than seniors for being closer to their university experience period or whether the most experienced professionals know more for having more clinical practice years. In addition, it should be taken into consideration that professionals with 30 years of experience or more have received information from the studies carried out in the 1980s, which have indicated cancer aspects that are similar to those found in current studies [30–32].

**Figure 1:**
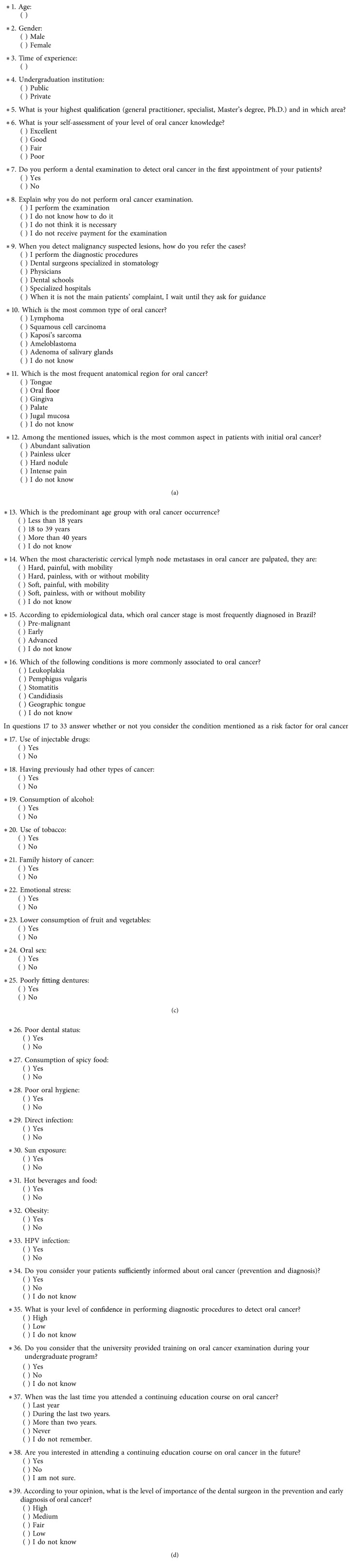
Questionnaire applied to assess oral cancer knowledge (Survey Monkey Brazil Internet Ltd.).

In Brazil, there are no studies that have assessed these differences justified by the time of experience with regard to oral cancer knowledge. Therefore, the objective of the present study was to assess dentists' knowledge about oral cancer by means of a literature-validated questionnaire (Figure 1) and to compare the knowledge level among two groups of professionals: junior or newly graduated (0 to 5 years of professional practice) versus senior dental clinicians (above 30 years of experience).

## 2. Materials and Methods

The present study was approved by the Research Ethics Committee of Paulista University (UNIP), São Paulo, Brazil (approval report 1,543,946-CAAE 54493716.8.0000.5512).

A cross-sectional observational study was carried out, where the database of the Regional Dentistry Council of São Paulo was accessed, which contained 28,671 listed professionals at the time of questionnaire submission, out of which 25,321 had their e-mails listed in their profile within the council database.

A questionnaire validated by Dib et al. [19] was modified and uploaded for online accessing using the Survey Monkey online platform (Survey Monkey Brazil Internet Ltd., São Paulo, Brazil) (Figure 1). The e-mails with the invitation to participate in the study along with the web link to access the questionnaire and a published consent form were distributed on July 2016 to the 25,321 listed dentists.

Out of all e-mails sent, 5,167 were not received due to outdated profile information or incorrect e-mail addresses in the profiles, and 20,154 e-mails were correctly received. After allowing one month for responses, the received data were fed into an Excel spreadsheet. The participation was anonymous, and no personal identification from the participants was registered.

The questionnaire consisted of 39 items divided into three parts (Figure 1). The first part covered the participants' general characteristics regarding their clinical practice related to the disease and interest in the topic. Values were not attributed to the responses in the first part. The second part addressed the knowledge about the clinical characteristics of oral cancer occurrence through six questions. Each question was worth one point. The third part consisted of 17 questions regarding risk factors, along with the question about oral cancer identification and diagnosis stage and time, totalling four points (Figure 1).

Grades were attributed to each participant according to their knowledge level. The applied criteria were A (excellent) for those who scored from 9 to 10 points; B (good) for those who scored from 7 to 8.99 points; C (regular) for those who scored from 5 to 6.99 points; and D (poor) for those who scored below 4.99 points.

The variables “age” and “seniority” (Figure 1) were categorized to perform the cross-tabulation of the questions and compared according to the junior and senior dental clinicians' knowledge level.

The statistical analysis was carried out in two stages. First, the univariate test was performed using Pearson's chi-square test with *α* = 0.20 to detect possible associations of the responses according to the time of formation. Subsequently, a multiple logistic regression analysis was performed with *α* = 0.05, with the dependent variable being the appropriate level of knowledge (A and B) in function of the independent variables (age, gender, time of experience, graduation institution, self-assessment of knowledge, level of confidence in performing diagnostic procedures, training at the university, qualification, and if attended a course on oral cancer) to obtain the odds ratios and the confidence intervals. The SPSS 22 statistical program was used (Statistical Package for Social Sciences, IBM^™^, Chicago, USA).

## 3. Results

A total of 20,154 e-mails were sent; however, only 477 of them were replied, representing a response rate of 2.36%. The participants were grouped according to practice seniority in order to compare 84 newly graduated dental clinicians with 105 senior dental clinicians. With this, the sample corresponded to 189 participants (Tables 1–6).

There was a statistical difference in the variable “gender” according to seniority in the comparison between the two groups (Table 1). The percentage of junior female dental clinicians was 78.6%, whereas the percentage of senior female dental clinicians was 57.1% (Table 1).

Regarding the knowledge level, there was a statistical difference according to the participants' seniority. Among the newly graduated dental clinicians, 19% obtained grade A (excellent), whereas only 6.7% of the senior dental clinicians obtained the same grade (Table 1).

The assessment of the variable “qualification” (Figure 1) showed that the results were also statistically different according to seniority (Table 1). Among the junior dental clinicians, 55.9% reported being general dental practitioners, 38.1% declared themselves specialists, and 6% had a Master's degree. On the other hand, among the senior dental clinicians, 56.2% reported being specialists, 21% were general dental practitioners, 15.2% had a Master's degree, and 7.6% held PhDs (Table 1). There was no statistical difference in the responses from both groups regarding the knowledge about the clinical characteristics of oral cancer (Table 2).

With regard to the risk factors of oral cancer development (Figure 1), there was a statistical difference between the two groups of professionals in the responses relating to “low consumption of fruit and vegetables,” “poor fitting of dentures,” “poor dental status,” “poor oral hygiene,” and “consumption of hot beverages and food” (Table 3).

Table 4 shows the response frequencies according to the factors related to attitudes toward oral cancer diagnosis and the perception about the topic according to the participants' seniority.

The multiple logistic regression analysis results indicated that the oral cancer knowledge of junior dental clinicians was 2.1 times higher in comparison to the senior dental clinicians' knowledge (OR = 2.1; 1.1–3.9 95% CI; *p*=0.024) (Table 6). In addition, it was found that the professionals who had graduated from public institutions were 2.3 times more aware about oral cancer (OR = 2.3; 1.2–4.3 95% CI; *p*=0.013). The participants who performed self-assessment and reported having satisfactory oral cancer knowledge (excellent or good) were 2.2 times more likely to have higher knowledge level (OR = 2.2; 1.2–4.2 95% CI; *p*=0.013) in comparison to the participants who reported regular or poor knowledge level (Table 6).

## 4. Discussion

Studies assessing dentists' knowledge, opinions, and practices relating to the prevention and early detection of oral cancer have been carried out in several countries [2, 4, 10, 11, 18, 20–25, 33–40]. The use of Internet and e-mails to obtain information has increased in recent years [2, 20, 41]. No articles have been found in Brazil with regard to the assessment of the dentists' oral cancer knowledge level considering and comparing their practice seniority, that is, newly graduated professionals versus senior professionals.

In contrast to the response rate observed in this study, in a Japanese study [20], which used the same electronic platform and sent 131 questionnaires, the response rate was 62.6%, represented by the 82 e-mails in response to the research. A Spanish study with 1,000 sent e-mails had 795 acknowledged as received and 340 (42.7%) responded questionnaires [2]. In contrast, another Brazilian study [27] sent 5,000 questionnaires via e-mail and the response rate was 1.4%, suggesting that the Brazilian professional population may be less partaking in scientific research, especially with respect to the elected method of data collection.

Therefore, it is natural to envision that Brazilian dentists have little interest in the subject. However, one possible explanation for the low response rate in the present study could be the excessive circulating advertising or spam and the ease with which they can be ignored or discarded [2]. However, the authors agree with López-Jornet et al. [2] when they say that this sort of communication is faster and easier to manage, in addition to being less costly. Therefore, new efforts and resources should be made for e-mails to be taken into consideration in future research so that important contents do not go unnoticed.

Nevertheless, despite these limitations of the low response rate, the number of participants (477) represents an expressive sample in comparison to the ones found in literature [2, 4, 10, 18, 20, 22–24, 27–29, 36, 42]. Therefore, the present study provides significant information about the knowledge of dentists in São Paulo that may contribute to new projects.

According to the dentists' obtained knowledge level grades, there was a statistical difference between newly graduated clinicians and senior dental clinicians (Table 1). Among junior dental clinicians, 19% obtained grade A (excellent) in comparison to 6.7% of senior dental clinicians. The results of the logistic regression analysis indicated that the knowledge level of junior dental clinicians was 2.1 times higher (OR = 2.1; 1.1–3.9 95% CI; *p*=0.024) (Table 6). This result is similar to that of other studies [4, 25].

Although there was a significant difference between the two groups, the data analysis allowed to observe that there are many concepts that are still not well-defined amongst professionals of both groups, showing that there is much to be discussed on means to stimulate oral cancer knowledge building.

In the question about the anatomical region of higher oral cancer prevalence (Figure 1), 45.5% of participants did not know the answer (Table 2). Rocha-Buelvas et al. [24] revealed that only a few professionals knew the most frequent locations of oral malignance. This is disturbing because if the professionals do not have the adequate knowledge about the most frequent locations of oral cancer development, then the injury may go unnoticed during a routine examination and, thus, the disease diagnosis may be delayed or ignored.

Our study revealed that one-third of the respondents do not know about regional metastases (Table 2), which coincides with a study conducted in New York, USA [21]; other studies found that less than 40% of dentists have reported that they palpated the patients' lymph nodes during the complete examination of oral cavities [11, 43]. These data highlight the need to improve the professionals' level of knowledge about clinical characteristics and cancer screening, giving that lymph node palpation often aids in the diagnosis of the disease during its asymptomatic stage.

The questions regarding the risk factors of the disease (tobacco, alcohol, and HPV) (Figure 1) were properly answered by the groups of professionals (Table 3), in opposition to a Japanese study [20], in which alcohol and HPV were poorly identified as risk factors for oral cancer. It is possible that the results of these studies are due to massive campaigns about the dangers of cigarettes and alcohol.

An interesting aspect was the controversy about the trauma of poor denture fitting (Figure 1), since 60.7% of junior clinicians and 93.3% of senior clinicians reported that it was a risk factor (Table 3), demonstrating that, despite the statistical difference, more than 60% of the professionals took this controversial issue into consideration. Although from the scientific perspective, the injuries caused by poor denture fitting do not cause cancer, these chronic injuries alter the oral environment and mask the symptoms, and initial lesions may not be properly diagnosed. Therefore, the professionals should eliminate these traumatic factors in the maintenance of oral health.

In the current study, 54% of respondents answered that poor oral hygiene (Figure 1) is a risk factor for oral cancer (Table 3). However, the role of poor oral hygiene is controversial, and this study corroborates with the observations from Oji and Chukwuneke [44], considering that only a major prospective study would provide appropriate information to scientifically clarify its impact in oral cancer genesis.

The low consumption of fruit and vegetables (Figure 1) was considered as a risk factor for oral cancer by 40.2% of professionals (Table 3). It is believed that eating fruit and vegetables may reduce the risk of cancer, including oral cancer, because they play an important part as a protective factor. Shivappa et al. [45] suggested a positive interaction between a proinflammatory diet with alcohol consumption and smoking in association with oral cancer. However, Dholam and Chouksey [46] found that a diet as a risk factor for oral cancer was not statistically significant. Moreover, this study agrees with Scully's [5] research that randomized clinical trials are needed to explore the effectiveness of dietary supplementation as chemoprevention to reduce the risk of oral cancer.

It is worth mentioning the importance appointed by the professionals regarding emotional stress (Figure 1). This issue was reported as a risk factor for oral cancer by 62.4% of dentists (Table 3). A recent study found an increased risk of oral cancer in patients who had suffered emotional stress. However, according to Dholam and Chouksey [46], emotional stress is a modern life symptom, and it may be responsible for delays in diagnosis due to work and family-related commitments, which probably generate patients' negligence toward their symptoms, but emotional stress would not be the core cause of oral cancer. Prospective studies with oral cancer patients would be necessary, excluding those who have scientifically proven risk factors, such as tobacco and alcohol consumption, and/or genetic factors, to show whether emotional stress alone could cause the disease.

The assessment of the variable “oral sex” (Figure 1) indicated a considerable number of positive responses, being a risk factor for 55.6% of professionals (Table 3). Nevertheless, these results may be a confounding factor because they probably associate oral sex with the possibility of HPV infection, which is strongly related to oral cancer [3, 7, 47]. Therefore, it is essential to provide patients with information about HPV and regarding the importance of preventive methods during sexual intercourse, in addition to the possibility of vaccination as a method to prevent virus infection.

Considering the attitudes for the diagnosis of suspected lesions (Figure 1), 17.9% of junior dental clinicians reported that they usually referred these cases to dental schools, compared to 2.4% of senior dental clinicians (Table 4). These results may be due to the fact that recent graduates feel more familiar with those institutions, possibly due to their recent undergraduation.

When asked about oral cancer screening training during the undergraduation (Figure 1), 70.2% of the junior clinicians reported having received training, compared to 43.8% of senior clinicians (Table 4). This means that almost 30% of the professionals were not properly trained, demonstrating that much needs to be improved in that aspect, considering the importance and seriousness of the matter. A study conducted in Spain [2] found that dentists, who were trained on oral cancer during their undergraduation, were more likely to agree that they had updated knowledge. This finding corroborates with the ones of the present study, since 66% of the participants that “rated themselves with satisfactory knowledge level” (excellent or good) reported that they had been trained for the examination of oral cancer during their undergraduation studies (*p*=0.002) (Table 5). In addition, logistic regression analysis indicated that they were 2.2 times more likely to have greater knowledge about the disease (OR = 2.2; 1.2–4.2 95% CI; *p*=0.013) (Table 6).

The logistic regression analysis indicated that dentists who graduated from public institutions had 2.3 times more knowledge about oral cancer in comparison to private institutions' graduates (OR = 2.3; 1.2–4.3 95% CI; *p*=0.013) (Table 6), demonstrating that specific studies on the analysis of the curriculum of public and private universities may be object of further research.

Considering participation in “continuing education courses on oral cancer” (Figure 1), 39.2% of the professionals had attended a course on oral cancer in the previous year or in the last two years (Table 4). This result is disturbing since the knowledge acquired during undergraduation tends to weaken with the absence of further knowledge support or updates [23]. Also, in the present study, 53% of the participants that reported satisfactory knowledge level (excellent or good) had attended a course on oral cancer in the last two years (*p* ≤ 0.001) (Table 5), coinciding with the study carried out by Hertrampf et al. [36], which found that the perceptions and practice relating to early detection of oral cancer had improved, particularly in the group of dentists that had attended further educational courses, emphasizing that these programs improved dental professionals' competence, findings in agreement with other studies [10, 23–25]. In Spain, the professionals who had benefited from continuing education courses were 3.5 times more likely to perform biopsies in suspicious lesions and twice as likely to give advice about alcohol consumption to patients [48], demonstrating the positive effect of further studies and professional updates.

Therefore, it is necessary that the professionals have greater interest in continuing education courses so that their knowledge and skills may be updated, contributing to the oral cancer prevention and minimizing practical failures regarding cancer screening, providing, when necessary, early disease detection.

The results of the present study demonstrated that although the junior dental clinicians had a knowledge level 2.1 times higher compared to senior dental clinicians (Table 6), there is still lack of knowledge about some topics related to risk factors and clinical characteristics of the disease.

Probably, as these results may be explained by the fact that the information obtained by newly graduated clinicians was more updated, or due to the lack of practice in the area, more experienced dentists were not interested in the subject. Further studies conducted with a larger number of professionals are required to confirm the results of this study.

## 5. Conclusion

Within the limitations of the study due to the low percentage of responses, we can conclude that, among the studied population, the newly graduated clinicians had a 2.1 times higher knowledge level in comparison to dentists who had more than 30 years of practice experience. However, when several factors regarding the knowledge of the risk factors and diagnostic procedures were individually assessed, the results indicated high rates of incorrect answers, demonstrating that there is room for further studies in the area and for oral cancer information activities. Therefore, oral cancer aspects must be emphasized so that more people, clinicians, and patients become interested in the topic. This goal may be achieved through clarification campaigns, dental school's program improvement, and the encouragement of professionals in attending continuing education courses for better qualification. New studies must be performed to compare our results.

## Figures and Tables

**Table 1:** Distribution of the number and percentages of responses regarding dentists' general characteristics according to responders' seniority.

Categorical	Variables	Dental clinicians		Total (%)	*p*
		Junior	Senior		
Grade obtained	A (excellent)	16 (19%)	7 (6.7%)	23 (12.2%)	**0.025** ^∗^
	B (good)	39 (46.4%)	44 (41.9%)	83 (43.9%)	
	C (regular)	20 (23.8%)	40 (38.1%)	60 (31.7%)	
	D (poor)	9 (10.7%)	14 (13.3%)	23 (12.2%)	
Gender	Female	66 (78.6%)	60 (57.1%)	126 (66.7%)	**0.002** ^∗^
	Male	18 (21.4%)	45 (42.9%)	63 (33.3%)	
Institution	Public	29 (34.5%)	44 (41.9%)	73 (38.6%)	0.300
	Private	55 (65.5%)	61 (58.1%)	116 (61.4%)	
Qualification	General practitioner	47 (55.9%)	22 (21%)	69 (36.5%)	**≤0.001** ^∗^
	Specialist	32 (38.1%)	59 (56.2%)	91 (48.1%)	
	Master's degree	5 (6%)	16 (15.2%)	21 (11.1%)	
	PhD	0 (0)	8 (7.6%)	8 (4.2%)	
		**84 (44.4%)**	**105 (55.6%)**	**189 (100%)**	

*Note.* ^∗^ *p* values lower than 0.05 indicate statistically significant results.

**Table 2:** Distribution of the number and percentages of responses to specific questions about oral cancer knowledge according to responders' seniority.

Variables	Categories	Dental clinicians		Total (%)	*p*
		Junior	Senior		
Most common cancer	Squamous cell carcinoma	57 (67.9%)	68 (64.8%)	125 (66.1%)	0.655
	Other	27 (32.1%)	37 (35.2%)	64 (33.9%)	
Most frequent anatomical region	Tongue	50 (59.5%)	53 (50.5%)	103 (54.5%)	0.215
	Other	34 (40.5%)	52 (49.5%)	86 (45.5%)	
Most common aspect in initial cancer	Painless ulcer	72 (85.7%)	90 (85.7%)	162 (85.7%)	1.000
	Other	12 (14.3%)	15 (14.3%)	27 (14.3%)	
Most common age group	More than 40 years old	75 (89.3%)	92 (87.6%)	167 (88.4%)	0.723
	Other	9 (10.7%)	13 (12.4%)	22 (11.6%)	
Most characteristic regional lymph node metastasis	Hard, painless, with or without mobility	58 (69%)	73 (69.5%)	131 (69.3%)	0.944
	Other	26 (31%)	32 (30.5%)	58 (30.7%)	
Diagnostic status in Brazil	Advanced	64 (76.2%)	87 (82.9%)	151 (79.9%)	0.256
	Other	20 (23.8%)	18 (17.1%)	38 (20.1%)	
Most common condition associated with cancer	Leukoplakia	62 (73.8%)	79 (75.2%)	141 (74.6%)	0.823
	Other	22 (26.2%)	26 (24.8%)	48 (25.4%)	
		**84 (44.4%)**	**105 (55.6%)**	**189 (100%)**	

*Note.p* values lower than 0.05 indicate statistically significant results; other = one of the incorrect answers.

**Table 3:** Distribution of the number and percentages of responses to specific questions addressing the knowledge about risk factors of oral cancer according to responders' seniority.

Variables	Categories	Dental clinicians		Total (%)	*p*
		Junior	Senior		
Injected drug use	Yes	29 (34.5%)	30 (28.6%)	59 (31.2%)	0.380
	No	55 (65.5%)	75 (71.4%)	130 (68.8%)	
Had other types of cancer previously	Yes	70 (83.3%)	83 (79%)	153 (81%)	0.456
	No	14 (16.7%)	22 (21%)	36 (19%)	
Alcohol consumption	Yes	81 (96.4%)	105 (100%)	186 (98.4%)	0.051
	No	3 (3.6%)	0 (0)	3 (1.6%)	
Tobacco consumption	Yes	84 (100%)	105 (100%)	189 (100%)	—
	No	0 (0)	0 (0)	0 (0)	
Family history of cancer	Yes	80 (95.2%)	100 (95.2%)	180 (95.2%)	1.000
	No	4 (4.8%)	5 (4.8%)	9 (4.8%)	
Emotional stress	Yes	47 (56%)	71 (67.6%)	118 (62.4%)	0.100
	No	37 (44%)	34 (32.4%)	71 (37.6%)	
Lower consumption of fruit and vegetables	Yes	26 (31%)	50 (47.6%)	76 (40.2%)	**0.020** ^∗^
	No	58 (69%)	55 (52.4%)	113 (59.8%)	
Oral sex	Yes	43 (51.2%)	62 (59%)	105 (55.6%)	0.280
	No	41 (48.8%)	43 (41%)	84 (44.4%)	
Poorly fitting dentures	Yes	51 (60.7%)	98 (93.3%)	149 (78.8%)	**≤0.001** ^∗^
	No	33 (39.3%)	7 (6.7%)	40 (21.2%)	
Poor dental status	Yes	37 (44%)	80 (76.2%)	117 (61.9%)	**≤0.001** ^∗^
	No	47 (56%)	25 (23.8%)	72 (38.1%)	
Consumption of spicy food	Yes	20 (23.8%)	36 (34.3%)	56 (29.6%)	0.117
	No	64 (76.2%)	69 (65.7%)	133 (70.4%)	
Poor oral hygiene	Yes	34 (40.5%)	68 (64.8%)	102 (54%)	**≤0.001** ^∗^
	No	50 (59.5%)	37 (35.2%)	87 (46%)	
Direct infection	Yes	9 (10.7%)	19 (18.1%)	28 (14.8%)	0.156
	No	75 (89.3%)	86 (81.9%)	161 (85.2%)	
Sun exposure	Yes	76 (90.5%)	86 (81.9%)	162 (85.7%)	0.094
	No	8 (9.5%)	19 (18.1%)	27 (14.3%)	
Hot beverages and food	Yes	34 (40.5%)	78 (74.3%)	112 (59.3%)	**≤0.001** ^∗^
	No	50 (59.5%)	27 (25.7%)	77 (40.7%)	
Obesity	Yes	14 (16.7%)	17 (16.2%)	31 (16.4%)	0.930
	No	70 (83.3%)	88 (83.8%)	158 (83.6%)	
HPV infection	Yes	71 (84.5%)	97 (92.4%)	168 (88.9%)	0.088
	No	13 (15.5%)	8 (7.6%)	21 (11.1%)	
		**84 (44.4%)**	**105 (55.6%)**	**189 (100%)**	

*Note.* ^∗^ *p* values lower than 0.05 indicate statistically significant results.

**Table 4:** Distribution of the number and percentages of responses about attitudes toward diagnosis of cancer and perception about this issue according to responders' seniority.

Variables	Categories	Dental clinicians		Total (%)	*p*
		Junior	Senior		
Self-assessment of knowledge	Excellent/good	46 (54.8%)	54 (51.4%)	100 (52.9%)	0.648
	Regular/poor	38 (45.2%)	51 (48.6%)	89 (47.1%)	
Performs cancer exam in the 1st appointment	Yes	66 (78.6%)	90 (85.7%)	156 (82.5%)	0.199
	No	18 (21.4%)	15 (14.3%)	33 (17.5%)	
Reason for not performing the exam	Performed the exam	67 (79.8%)	88 (83.8%)	155 (82%)	0.551
	I do not know how to do it	8 (9.5%)	11 (10.5%)	19 (10.1%)	
	I do not think it is necessary	6 (7.1%)	5 (4.8%)	11 (5.8%)	
	I do not receive fees	3 (3.6%)	1 (1%)	4 (2.1%)	
Referral of suspicious lesions	Stomatology	53 (63.1%)	73 (69.5%)	126 (66.7%)	**0.007** ^∗^
	Myself	13 (15.5%)	19 (18.1%)	32 (16.9%)	
	Dental school	15 (17.9%)	3 (2.9%)	18 (9.5%)	
	Specialized hospital	2 (2.4%)	6 (5.7%)	8 (4.2%)	
	Physician	1 (1.2%)	4 (3.8%)	5 (2%)	
Confidence level	High	26 (31%)	40 (38.1%)	66 (34.9%)	0.407
	Low	55 (65.5%)	59 (56.2%)	114 (60.3%)	
	I do not know	3 (3.6%)	6 (5.7%)	9 (4.8%)	
Training at the university	Yes	59 (70.2%)	46 (43.8%)	105 (55.6%)	**≤0.001** ^∗^
	No	25 (29.8%)	55 (52.4%)	80 (42.3%)	
	I do not know	0 (0)	4 (3.8%)	4 (2.1%)	
Attended a course on oral cancer	Last year	17 (20.2%)	13 (12.4%)	30 (15.9%)	**0.006** ^∗^
	Two years ago	24 (28.6%)	20 (19%)	44 (23.3%)	
	More than two years ago	19 (22.6%)	52 (49.5%)	71 (37.6%)	
	Never	14 (16.9%)	12 (11.4%)	26 (13.8%)	
	I do not remember	10 (11.9%)	8 (7.6%)	18 (9.5%)	
		**84 (44.4%)**	**105 (55.6%)**	**189 (100%)**	

*Note.* ^∗^ *p* values lower than 0.05 indicate statistically significant results.

**Table 5:** Distribution of the number and percentages of responses relating to the dentists' general characteristics according to their self-assessment of oral cancer knowledge.

Variables	Categories	Self-assessment of the level of knowledge about oral cancer		Total (%)	*p*
		Satisfactory	Unsatisfactory		
Institution	Public	43 (43%)	30 (33.7%)	73 (38.6%)	0.190
	Private	57 (57%)	59 (66.3%)	116 (61.4%)	
Time of experience	Junior dental clinicians	46 (46%)	38 (42.7%)	84 (44.4%)	0.648
	Senior dental clinicians	54 (54%)	51 (57.3%)	105 (55.6%)	
Qualification	General practitioner	35 (35%)	34 (38.2%)	69 (36.5%)	0.648
	Graduated^1^	65 (65%)	55 (61.8%)	120 (63.5%)	
Training at the university	Yes	66 (66%)	39 (43.8%)	105 (55.6%)	**0.002** ^∗^
	No/I do not know	34 (34%)	50 (56.2%)	84 (44.4%)	
Attended a course on oral cancer	Two years ago	53 (53%)	21 (23.6%)	74 (39.2%)	**≤0.001** ^∗^
	More than two years ago/never	47 (47%)	68 (76.4%)	115 (60.8%)	
Grades obtained	A-B (excellent/good)	67 (67%)	39 (43.8%)	106 (56.1%)	**≤0.001** ^∗^
	C-D (regular/poor)	33 (33%)	50 (56.2%)	83 (43.9%)	
		**100 (52.9%)**	**89 (47.1%)**	**189 (100%)**	

*Note.* ^∗^ *p* values lower than 0.05 indicate statistically significant results. ^1^The “Graduated” category refers to the participants that reported having specialization, Master's degree, and/or PhD.

**Table 6:** Association of the general characteristics and clinical practice of the dentists relating to the level of knowledge about oral cancer according to attributed grades (A = excellent; B = good).

Characteristics	Categories	Grades obtained (A = excellent; B = good)		
		Number (%)	OR (95% CI)	*p*
Self-assessment of knowledge	Satisfactory	100 (52.9%)	2.2 (1.2–4.2)	**0.013** ^∗^
	Unsatisfactory	89 (47.1%)		
Time of experience	Junior dental clinicians	84 (44.4%)	2.1 (1.1–3.9)	**0.024** ^∗^
	Senior dental clinicians	105 (55.6%)		
Graduation institution	Public	73 (38.6%)	2.3 (1.2–4.3)	**0.013** ^∗^
	Private	116 (61.4%)		
Attended a course on oral cancer	Two years ago	74 (39.2%)	1.5 (0.8–2.9)	0.234
	More than two years ago or never	115 (60.8%)		

*Note.* ^∗^ *p* values lower than 0.05 indicate statistically significant results. The time when the responder attended a course on oral cancer was an adjustment variable for the multiple logistic regression analysis.
